# Editorial: Brain arteriovenous malformations: cerebrovasculature behaving badly

**DOI:** 10.3389/fnhum.2023.1212184

**Published:** 2023-06-07

**Authors:** Lorelei D. Shoemaker, Richard Daneman, Marcus A. Stoodley

**Affiliations:** ^1^Department of Neurosurgery, Stanford School of Medicine, Stanford, CA, United States; ^2^Department of Neurosciences, Department of Pharmacology, School of Medicine, University of California, San Diego, San Diego, CA, United States; ^3^Faculty of Medicine, Health and Human Sciences, Macquarie University, Sydney, NSW, United States

**Keywords:** cerebrovascular disease, hemorrhage, endothelial cells, neurovascular unit (NVU), Hereditary Hemorrhagic Telangiectasia (HHT), arteriovenous malformations (AVMS), neuroinflammation, Sturge-Weber syndrome

Brain arteriovenous malformations (bAVMs) are a rare disease, consisting of a nest of abnormal arteries and veins, and lacking a capillary bed. Diagnosis is often made when patients present with seizures, migraines or, more seriously, hemorrhage, although identification of asymptomatic AVMs is occurring more frequently, owing to increased imaging. AVMs are heterogeneous and vary in terms of blood flow, age of onset/treatment, size, venous drainage, and brain location. With no clear standard of care, treatment is on a case-by-case basis, with limited ability to predict hemorrhage risk. Guided by the specific features of the AVM, treatment proceeds with increasing invasiveness, consisting of any combination of monitoring, embolization, radiosurgery, to surgical removal. Complete AVM resolution often requires years, with risk of hemorrhage – and increasing patient stress - throughout. Understanding hemorrhage risk for symptomatic and asymptomatic AVMs would present a breakthrough in treatment decisions. However, AVM disease biology remains unclear, hindering improvements in diagnosis and treatment. Our goal for this Research Topic is to highlight advances in the field of AVMs, and to suggest where gaps remain.

The blood vessels and myriad cell types in the brain share an intimate relationship during development, in the adult, and in disease. Within the neurovascular unit (NVU), endothelial cells (ECs), pericytes, vascular smooth muscle cells (SMCs), astrocytes, microglia, and neurons, together with a complex basement membrane, interact to establish the blood-brain barrier (BBB) and normal brain function. The AVM microenvironment is complex and involves interplay between the cells present in the NVU (Shabani et al.). There are likely transitional or immature cell types present, involved in pathogenic processes such as endothelial-to-mesenchymal transition (EndMT). Mural cells - the SMCs and pericytes that normally stabilize the vasculature - are also affected. In a rodent model of Hereditary Hemorrhagic Telangiectasia (HHT), EC-specific deletion of Rbpj, a mediator of Notch signaling, results in altered pericyte coverage, highlighting EC and pericyte communication in neurovascular disease (Selhorst et al.). A compromised BBB and/or shear stress drives an inflammatory response, with the presence of macrophages, neutrophils, and T lymphocytes (Shabani et al.). Understanding how these cells interact in a pathological environment may hold clues to disease mechanisms, initiating event(s), and probability of hemorrhage.

Mechanistic insight into the pathogenesis of AVMs has focused on Notch, SMADs, VEGF, TGFβ, BMPs, KRAS, microRNAs and somatic mutations, among others. Interestingly, many of the signaling pathways implicated in AVMs are shared in cancer, including PI3K/AKT/mTOR, RAS/MAF/MEK, and KRAS. Re-purposing FDA-approved cancer drugs targeting angiogenesis and inflammation has promise in AVM research (Mansur and Radovanovic). Ultimately, there is likely no singular driver of the pathology, but rather a complex interplay amongst these mechanisms, underlining the challenges in designing treatments.

Approximately 95% of bAVMs are sporadic but can also occur as a part of genetic syndromes. HHT is characterized by various mutations in the transforming growth factor β (TGFβ) signaling pathway, with ENG and ACVRL1 genes accounting for ~90% of the cases (Drapé et al.). Importantly, genotype does not equate to phenotype, as each HHT patient has unique clinical features. Vascular malformations are also a component of Sturge-Weber syndrome, associated with somatic mutations in the GNAQ gene (Van Trigt et al.). Is there overlap with other vascular anomalies and AVMs? Given the heterogeneity of malformations, accurate molecular classification is crucial to understanding the pathophysiology, developing therapeutic targets, and guiding treatment (Mansur and Radovanovic; Drapé et al.).

Is this achievable? Biopsies are clearly not an option for bAVMs, but there has been some success in defining the molecular landscape in biofluids such as blood (Mansur and Radovanovic) and cerebrospinal fluid (Wichmann et al.). This goal remains distant, given the technical challenges of developing a “biomarker matrix” to identify and characterize relatively small AVMs amidst the ~100,000 km of vasculature in the body.

Animal models of AVMs have provided valuable insight into vascular malformations (Selhorst et al.). Rodent and zebrafish models not only highlight therapeutic targets but also provide systems amenable to hypothesis testing and molecular manipulation (Drapé et al.). An important consideration is how these largely genetic models compare with human AVMs. The clear advantage to human tissue and models is the more direct translation to human AVMs. In practice however, working with human tissue/models is challenging and there remains space to expand research tools to multidimensional cell models, vascularized brain organoids, and iPSC technologies (Van Trigt et al.).

Treatment goals are clear: low complication and mortality rates, and efficacy. Hemorrhage risk is the most potent driver of the treatment plan but clinicians cannot evaluate this to choose between conservative and aggressive management. This creates tremendous uncertainty in what the future holds for AVM patients. Improved diagnostic imaging has the potential to evaluate AVM flow and BBB leakage and to assist in estimating hemorrhage risk but is still in its early stages.

Conventional treatments are inadequate and often have detrimental effects. For instance, radiosurgery may result in damage to otherwise healthy tissue (Ung et al.). Embolization (endovascular therapy or EVT) is successful as a first line treatment in another rare vascular anomaly (Zhou et al.) but is an adjunct treatment prior to radiosurgery or surgery for AVMs. EVT may be suitable as a curative treatment for specific AVMs but this is controversial. In addition, the presence of other vascular anomalies complicates the treatment of AVMs (Wang et al.).

AVMs and cancer share many signaling pathways, opening the door to a tremendous arsenal of FDA-approved cancer drugs (Mansur and Radovanovic). With accurate molecular definitions of AVMs, therapeutics may be the least invasive and most precise, although the question remains of the impact these therapeutics will have on existing AVMs. Finally, development of treatments for the complications of AVMs, such as hemorrhagic stroke, is necessary (Dou et al.).

This Research Topic highlights the progress in understanding AVM microenvironment and mechanisms, molecular classification, development of models, and diagnosis and treatment. There remains a real need for hemorrhage-risk evaluation and for novel anti-angiogenesis and vascular targeting therapies. This effort will require a multidisciplinary approach, from basic research to patient care.

## Author contributions

LS, RD, and MS wrote and edited the manuscript. All authors contributed to the article and approved the submitted version.

